# Iridaaromatics via Methoxy(alkenyl)carbeneiridium Complexes

**DOI:** 10.3390/molecules26154655

**Published:** 2021-07-31

**Authors:** Maria Talavera, Sandra Bolaño

**Affiliations:** 1 Department of Chemistry, Humboldt–Universität zu Berlin, Brook-Taylor-Str. 2, 12489 Berlin, Germany; talaverm@hu-berlin.de; 2 Departamento de Química Inorgánica, Universidade de Vigo, Campus Universitario, 36310 Vigo, Spain

**Keywords:** metallaaromatics, iridium, PAHs

## Abstract

This review describes the development of a versatile methodology to synthesize polycyclic metallaaromatic hydrocarbons based on iridium, as well as the studies that helped us to determine and understand what is required in order to broaden the scope and the selectivity of the methodology and stabilize the complexes obtained. This methodology aims to open the door to new materials based on graphene fragments.

## 1. Introduction

The interest of graphene lies in its structure and unusual physical properties, which turn polycyclic aromatic hydrocarbons (PAHs) and nanographenes, small graphene fragments, into the subject of several research in the last decades [1]. Studying PAHs allows for correlating their geometrical and electronic structure with their properties, which can then be applied to larger structures. It has been found that the introduction of defects or irregularities in the structure of PAHs modifies their properties and consequently broadens their field of application. Among these defects, the most common are deviations from planarity due to edges, the introduction of non-six-member rings, doping with heteroatoms or the inclusion of substituents for their functionalization [2,3,4,5].

The introduction of these irregularities in PAHs can lead to new materials improving results and even opening up new applications with respect to their non-defective analogues. With this in mind, over the last decade our research group has focused its interest on the development of new PAHs by incorporating a transition metal in order to create polycyclic metallaaromatic hydrocarbons (PMAHs) by replacing a CH unit with a transition metal. These new systems are considered to be doped PAHs and could potentially introduce a slight deviation of the plane. This has led us to develop a synthetic and versatile methodology that allows us to obtain PMAHs with different structures and sizes, as this is the key to obtaining new and improved properties for their application in the field of materials.

In this review, we focus on the studies which led us to the successful development and understanding of a methodology for synthesizing iridium based PMAHs.

## 2. Methoxy(alkenyl)carbeneiridium Complexes

**Scheme 1:**
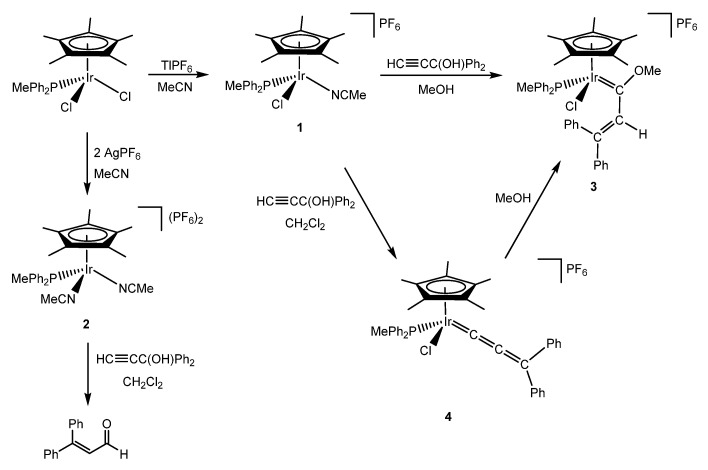
Synthesis of the acetonitrile complexes **1** and **2** and their reactivity towards propargylic alcohols.

The synthesis of methoxycarbenes is a well-known process which is achieved by the nucleophilic addition of methanol to a vinylidene or allenylidene complex [6,7]. One useful methodology to obtain these products is the activation of terminal alkynes and propargylic alcohols with a suitable precursor. In this regard, complexes with labile ligands, such as ethylene, cyclooctadiene or acetonitrile, are convenient starting materials [8]. Therefore, the acetonitrile complexes [IrCp*Cl(NCMe)(PPh_2_Me)]PF_6_ (**1**) and [IrCp*(NCMe)_2_(PPh_2_Me)](PF_6_)_2_ (**2**) obtained from [IrCp*Cl_2_(PPh_2_Me)] (Scheme 1) promised to be suitable precursors. It was observed that the chosen salt for the abstraction of the chlorido ligand played a key role in the product selectivity. Therefore, AgPF_6_ promotes a double methathesis (independent of the stoichiometry) while TlPF_6_ selectively leads to the monoacetonitrile derivative [9,10].

The monoacetonitrile complex **1** reacted with 1,1-diphenyl-2-propyn-1-ol in methanol, yielding the expected methoxy(alkenyl)carbeneiridium complex [IrCp*Cl{=C(OMe)CH=CPh_2_}(PPh_2_Me)]PF_6_ (**3**). When the reaction was performed in dichloromethane, the formation of an unstable allenylidene complex **4** was observed, which by addition of methanol yielded, as proposed, complex **3** (Scheme 1) [9].

Interestingly, the bisacetonitrile complex **2** was capable of catalytically activating propargylic alcohols in order to produce the corresponding aldehydes through a Meyer–Schuster rearrangement, instead of the expected organometallic derivative (Scheme 1). The mechanism proposed for the reaction involved the initial formation of a vinylidene complex which underwent an intramolecular nucleophilic addition of the hydroxo group to the *α*-carbon of the vinylidene [10]. This mechanism excluded the formation of an allenylidene complex and, therefore, hampered the nucleophilic attack of methanol to form methoxy(alkenyl)carbenes.

**Scheme 2:**
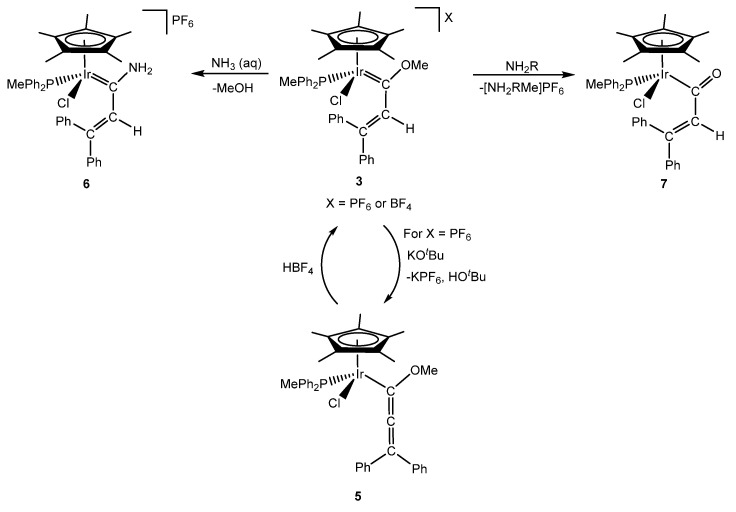
Reactivity of the methoxy(alkenyl)carbeneiridium complex **3**.

The reactivity of complex **3** towards strong bases and amines was studied. Thus, an excess of KO*^t^*Bu deprotonated the alkenyl moiety leading to the neutral methoxyallenyl complex [IrCp*Cl{C(OMe)=C=CPh_2_}(PPh_2_Me)] (**5**) (Scheme 2). This reaction is reversible, and the addition of HBF_4_ regenerated cation complex **3** [9]. On the other hand, reactivity with amines did not provide the expected results. It is known that primary or secondary amines can react with alkoxycarbenes to provide aminocarbenes through an aminolysis reaction [11,12,13]. However, this reaction only took place when an aqueous ammonia solution was used and the aminocarbene complex [IrCp*Cl{=C(NH_2_)CH=CPh_2_}(PPh_2_Me)]PF_6_ (**6**) could be isolated (Scheme 2). Instead, reactivity with amines underwent a heterolytic cleavage of the carbon(sp^3^)-oxygen bond to generate the acyl complex [IrCp*Cl{C(O)CH=CPh_2_}(PPh_2_Me)] (**7**) and the release of a mixture of methylammonium salts (Scheme 2) [9]. DFT calculations were performed in order to explain the different reactivity, and the presence of an equilibrium between electronic, steric and cooperative effects was revealed [14]. Calculations showed that the aminolysis reaction is not the dominant reaction with these particular alkoxycarbene complexes.

## 3. Iridanaphthalene Complex and Its Evolution

**Scheme 3:**
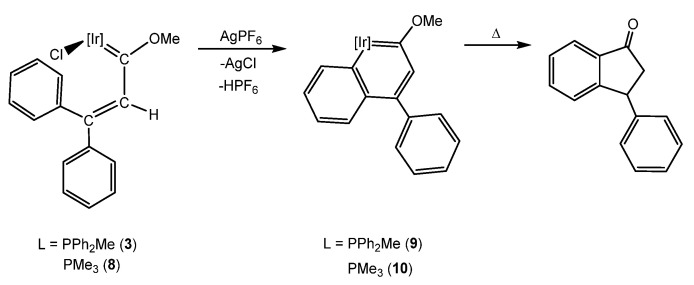
Synthesis and evolution of the iridanaphthalene complexes **9** and **10**. [Ir] = [IrCp*L]PF_6_.

Besides the unexpected chemistry displayed by complex **3** towards amines, the complexes [IrCp*Cl{=C(OMe)CH=CPh_2_}(L)]PF_6_ (L = PPh_2_Me (**3**), PMe_3_ (**8**)) [15] have been proved to be active towards orthomethalation of a phenyl substituent of the methoxy(alkenyl)carbene ligand. Thus, abstraction of the chlorido ligand, in complexes **3** and **8**, by addition of a silver salt, promoted the intramolecular C–H bond activation of a phenyl substituent of the carbene ligand, leading to the formation of a new Ir–C bond resulting in two new iridanaphthalene complexes, **9** and **10**, respectively, bearing a phenyl substituent in the iridanaphthalene moiety (Scheme 3) [16].

Complexes **9** and **10** represented the third example of metallanaphthalene complexes described, and the second with iridium as the transition metal [17,18]. Previously, Paneque et al. had reported the synthesis of the first iridanaphthalene complex through an oxidative ring contraction reaction of a benzannelated iridacycloheptatriene complex [18]. The X-ray structure of complexes **9·BPh_4_**, **10** and **10·BPh_4_** present C–C distances between 1.35 and 1.47 Å, comparing well with those of naphthalene, suggesting that the delocalized π-bonding extends over both rings. In these structures, the iridium atom is displaced from the nearly co-planar carbon atoms by 0.066(5) and 0.176(9) Å for **9·BPh_4_** and **10·BPh_4,_** respectively [16]. The values of the iridanaphthalene complexes with BPh_4_ as counteranion are significantly smaller than those reported in the literature on other iridanaphthalene complexes, which are very similar to the value observed for **10** (0.689(5) Å) [18,19].

In the study of metallaaromatic complexes, X-ray diffraction and NMR spectroscopy are the most widely used techniques, as they provide information about the structure and nature of these complexes [20]. In complexes **9** and **10**, two features are determined in order to confirm the formation of the cycle: (i) the absence of the proton resonance of the activated C–H bond in the ^1^H NMR spectra, and; (ii) the deshielding together with the C,P coupling in the carbon resonance (*δ* 155 ppm, ^2^*J*_C-P_ = 10 Hz) at the ^13^C{^1^H} NMR spectra when bonded to the iridium atom [16].

**Scheme 4:**
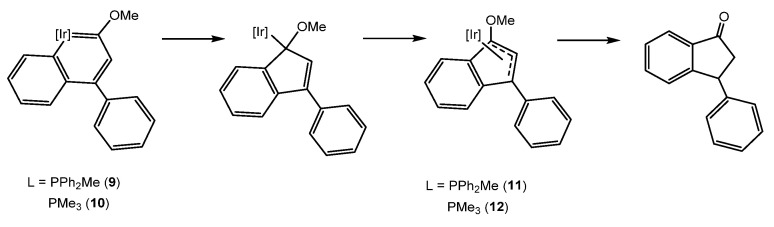
Mechanism proposal for the formation of 3-phenylindanone from complexes **9** and **10**. [Ir] = [IrCp*L]PF_6_.

As has happened in many metallaaromatic systems, **9** and **10** resulted in the presentation of a low thermal stability. While the literature described the rearrangement of the iridaaromatic complexes into the thermodynamically stable cyclopentadienyl derivatives [21,22], complexes **9** and **10** evolved into 3-phenylindanone when heat was applied (Scheme 3) [16]. Different experiments conducted in order to elucidate the mechanism of this transformation have allowed for the observation of the expected indenyl complexes [IrCp*{η^3^-(C_9_H_5_)(OMe)(Ph)}(L)]PF_6_ (L = PPh_2_Me (**11**), PMe_3_ (**12**)) as reaction intermediates. Therefore, the iridanaphthalene complexes **9** and **10** may undergo carbene migratory insertion, yielding *η^1^* indenyl complexes which would evolve to the *η^3^* intermediates **11** and **12** through a hapticity switch. Finally, the heterolytic cleavage of the O-CH_3_ bond would occur and hydrogenation of the indenone would take place in order to yield the final 3-phenylindanone (Scheme 4). In order to find the hydrogen source, a reaction was also performed in deuterated solvents as well as in strictly water-free conditions. These studies suggested the involvement of the methyl group in the hydrogenation reaction [16].

## 4. Effect of the *γ*-Carbon Substituents

**Scheme 5:**
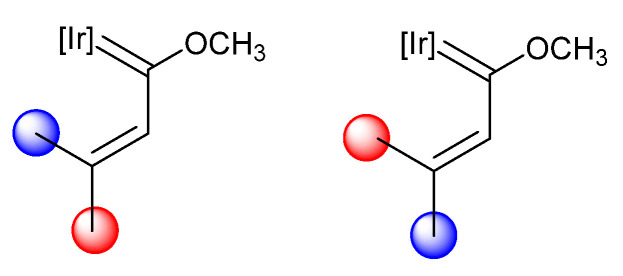
Possible *E* and *Z* isomers in methoxy(alkenyl)carbeneiridium complexes. [Ir] = [IrCp*Cl(PMe)_3_]^+^.

The understanding and control of the synthetic methodology, with methoxy(alkenyl)carbeneiridium complex as the starting material, will allow for the accessing of different and stable PMAHs. Thus, it was expected that the final product would depend on the substituents at a *γ* spatial arrangement of the precursor methoxy(alkenyl)carbeneiridium and, therefore, the key would be in the choice of the propargylic alcohol. In order to gain a better insight into the effect of the *γ* carbon substituent, methoxy(alkenyl)carbene complexes with two different substituents have been described, which may result in a mixture of the *E* and *Z* isomers (Scheme 5).

**Scheme 6:**
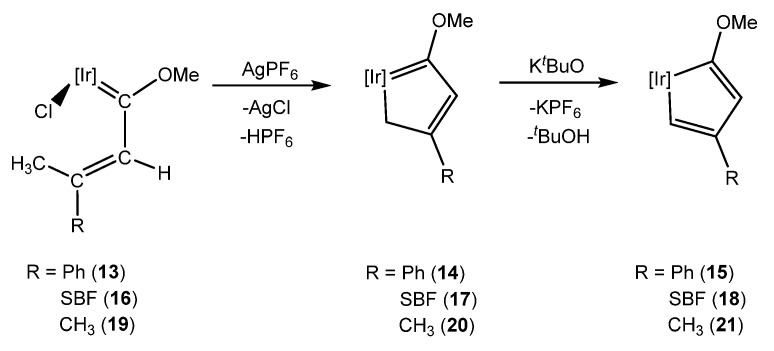
Formation of iridacyclopentadiene complexes. [Ir] = [IrCp*(PMe_3_)]PF_6_.

When the substituents on the *γ* carbon are a methyl and phenyl group (**13**), only the *E* isomer was formed, as the methyl group is located close to the iridium atom. With this spatial arrangement, AgPF_6_ treatment leads to C–H bond activation of the methyl group, obtaining the iridacyclopenta-1,3-diene complex **14** (Scheme 6). Complex **14** can react with a base in order to yield an iridacyclopenta-2,4-diene complex **15** [23]. The same behavior was observed at complex **16**, where the phenyl group was substituted with a spirobifluorene moiety (SBF) and complexes **17** and **18** were obtained (Scheme 6) [24]. Likewise, complex **19**, bearing two methyl groups, also performed the C–H bond activation, yielding **20**. After, the treatment of **20** with K*^t^*BuO, the iridacyclopentadiene complex **21** was formed (Scheme 6). However, two hydrogen atoms on *γ* carbon did not afford a stable iridacyclobutadiene complex [23].

To better understand the importance of the isomerism at the methoxy(alkenyl)carbene for the synthesis of iridaaromatic complexes, DFT calculations were performed (B3LYP level). Thus, in complexes **13** and **16**, the calculations predicted a single product: the *E* isomer. This was due to a higher stability of the *E* isomers over the *Z* isomers in these complexes with a difference of 3.94 kcal/mol and 4.85 kcal/mol, respectively. However, if the methyl group in **16** was substituted with a phenyl group, the ΔG would be 0.70 kcal/mol which would allow for the formation of small amounts of the *Z* isomer and therefore a mixture of iridaaromatic complexes [24]. These results showed that the spatial arrangement is crucial for the methodology and that the preference of one isomer over another is clearly influenced by the lower steric hindrance.

**Scheme 7:**
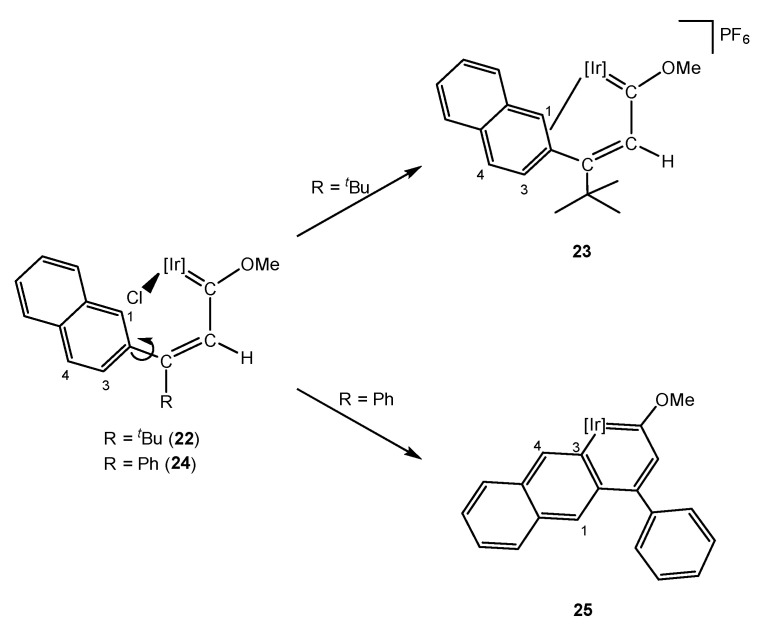
Activation of the methoxy(alkenyl)carbene complexes **22** and **24** yielding the *η*^2^ complex **23** and the iridaaromatic complex **25**, respectively. [Ir] = [IrCp*(PMe_3_)]PF_6_.

It was not possible, in the above examples, to establish the effect that an aliphatic substituent of the *γ* carbon may have on the formation of the corresponding metallaaromatic compound if, instead of obtaining the *E* isomer, one had the *Z* isomer. Thus, the methoxy(alkenyl)carbene **22** bearing a 2-naphtyl and a *tert*-butyl ligand was synthesized. Due to the steric hindrance of the *tert*-butyl group, only the *E* isomer was observed, which would facilitate the C–H bond activation of the aromatic substituent. However, treatment with silver salt did not provide the expected iridaaromatic complex, and instead the *η*^2^ complex **23** was identified (Scheme 7) [25]. Although complex **23** might be the intermediate for the formation of the iridaaromatic complex, further evolution to the corresponding iridaphenantrene was not observed. Notably, when the *tert*-butyl substituent in **22** was replaced by a phenyl group, the C–H bond activation took place at the methoxy(alkenyl)carbene **24** and the corresponding iridaaromatic compound **25** was obtained (Scheme 7).

**Figure 1:**
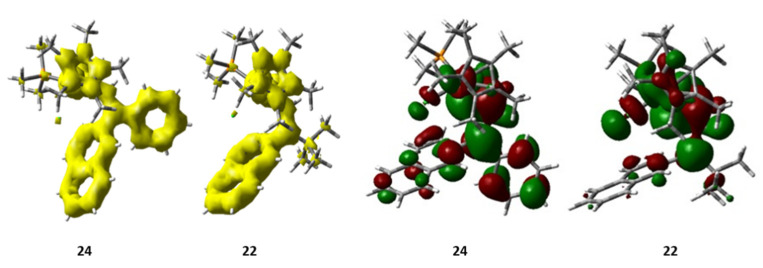
ACID representations (left) and LUMO orbitals (right) of complexes **22** and **24**.

Fukui indices determined that C1 was the most reactive position in **22**, leading to the unique formation of **23**, despite the free rotation of the naphthalenyl substituent. In contrast, complex **24** presented the higher nucleophilic Fukui index for C4, which was too far away from the iridium nucleus (Ir-C4 distance = 5.08 Å) leading to the activation of C3 which resulted in an iridaanthracene compound, **25** (see also Section 5.3). In addition, DFT calculations (B3LYP level) determined that the C1–H bond activation of **22** to give an iridaphenantrene complex would not be favored by 2.80 kcal/mol, possibly due to the steric hindrance of the final product. However, the effect of the *tert*-butyl moiety was further determined by comparison with complex **24 [25]**. In order to analyze electronic delocalization, different methodologies exist [26,27,28], including the anisotropy of the induced current density (ACID). ACID calculations showed that the electron density in **24** was delocalized along the whole system, contrary to what was observed in **22**. Similarly, the LUMO orbital for **22** was less widespread throughout the molecule than in **24** (Figure 1) and the HOMO-LUMO gap was bigger, supporting a low reactivity of the *tert*-butyl derivative. These findings endorsed the high influence of the electron density delocalization on the formation of this family of iridaaromatic complexes, which would be, therefore, promoted by second substituents such as aromatic rings.

## 5. Stability of the Metallaaromatic Systems

Besides establishing the requirement of two aromatic substituents at the *γ* carbon of the methoxy(alkenyl)carbene ligand, it is also important to analyze the effect of the metal, in order to study different topologies and improve the stability of these metallaaromatics by incorporating different substituents in the aromatic cycle.

### 5.1. Effect of the Aromatic Ring Substituents

The low thermal stability of the iridanaphthalene complexes **9** and **10** (see Section 3) reduces their possible applications in materials sciences. Therefore, it is important to assess the effect of different substituents in the phenyl rings of the iridaaromatic moiety in order to enhance their stability. While Haley and co-workers reported the higher tendency to the rearrangement of iridabenzene complexes when they bear bulky ligands [21,22], our group focused on the electronic effects on the stability of the iridanaphthalene system [29].

**Scheme 8:**
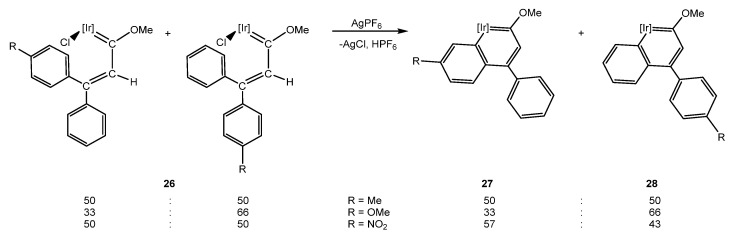
Formation of complexes **27** and **28** from *Z/E* isomers of **26**. [Ir] = [IrCp*(PMe_3_)]PF_6_.

Thus, the propargylic alcohols HC≡C(OH)(Ph)(*p*-R-C_6_H_4_) with R = NO_2_, OMe or CH_3_ where chosen in order to assess the electronic effect of such substituents on the formation of the substituted 3-phenylindanone derivatives. The similar steric hindrance between the non-substituted and the nitro- or methyl-substituted phenyl rings did not allow for obtaining selectively a unique methoxy(alkenyl)carbeneiridium isomer. Consequently, the C–H bond activation reaction of the mixture of the *E*- and *Z*-isomers **26·NO_2_/CH_3_** gave rise to their respective iridanaphthalenes **27** and **28** (Scheme 8). However, when one of the aromatic rings bears a methoxy group, the ratio between the *E*- and *Z*- isomers is not proportional, resulted in obtaining the *E*-isomer ***E*-26·OMe** in higher amounts, which may be due to electronic effects rather than the steric hindrance due to their location in a remote position (Scheme 8).

**Scheme 9:**
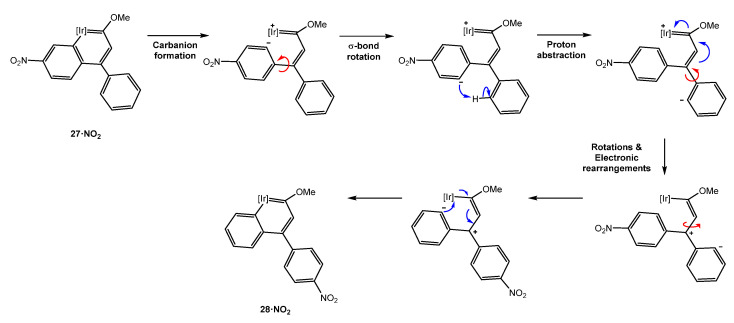
Isomerization mechanism of **27·NO_2_** into **28·NO_2_**. [Ir] = [IrCp*(PMe_3_)]PF_6_.

As happened for **10** these mixtures of iridanaphthalene complexes are not stable in a solution evolving to the indanone derivatives [29]. The study of their evolution established a direct correlation between the electronic properties of the groups in the iridanaphthalene moiety and the final indanone, where **27·OMe** possessing a π-donor group (-OMe) at position 6 of the metallanaphthalene backbone proved to be the most stable iridanaphthalene. On the other hand, the nitro group is an electron-withdrawing group through resonance and induction which facilitates the isomerization of complex **27·NO_2_** into **28·NO_2_** when located in position 6 of the naphthalene ring. This process was proposed to take place through the initial formation of a carbanion from the complex **27·NO_2_**, followed by a proton abstraction right after a rotation over the σ bond between C_γ_ and the substituted phenyl ring. Then, the rotations and electronic rearrangements would yield complex **28·NO_2_** (Scheme 9) [29].

### 5.2. Effect of the Transition Metal

In spite of the broad range of metals used to synthesized metallabenzenes [30], their higher analogues have been mainly described with iridium [18,19,31,32,33] together with an example of osmium [17] and ruthenium [34]. However, rhodium, the iridium congener, has been never found to form a metallabenzene complex. This fact suggests that not only the electronic properties of the metal play a role in the synthesis of metallaaromatic complexes but also the steric ones.

**Scheme 10:**
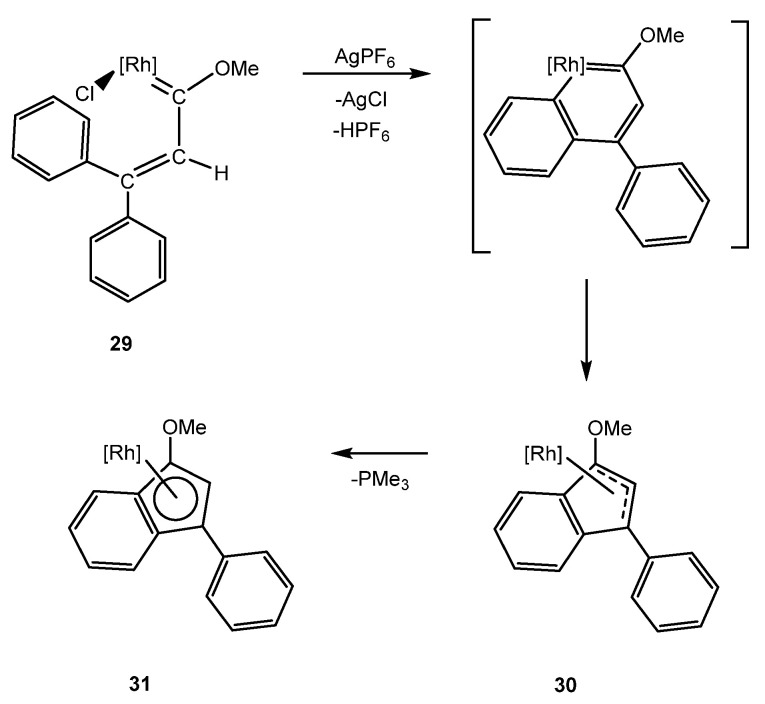
Implementation of the methodology using rhodium as transition metal. [Rh] = [RhCp*(PMe_3_)]PF_6_.

In order to study the effect of the transition metal in the methodology developed for the synthesis of iridanaphthalene complexes, the methoxy(alkenyl)carbene rhodium complex **29**, analogous to **3**, was synthesized from the corresponding monoacetonitrile complex. However, treatment of complex **29** with AgPF_6_ did not afford the expected rhodanaphthalene, producing instead the *η*^3^ complex **30** together with a 15% of the *η*^5^ complex **31**, due to the hapticity change of the indenyl ligand [35]. The formation of **30** resembles the evolution observed in the iridanaphthalene complexes **9** and **10**, which would suggest the formation of a very unstable rhodanaphthalene complex as intermediate (Scheme 10).

**Scheme 11:**
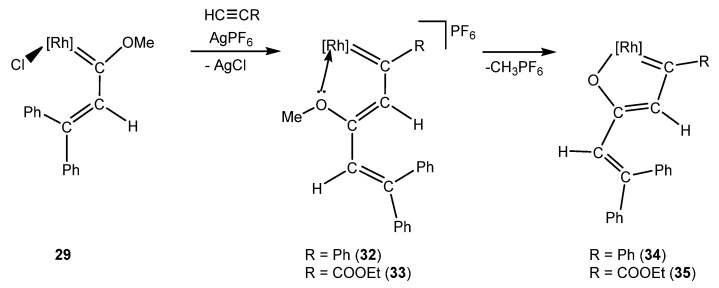
Effect of the involvement of alkynes in the studied methodology. [Rh] = [RhCp*(PMe_3_)]PF_6_.

Although the rhodanaphthalene complexes were not possible to stabilize, the synthesis of other family of metallaaromatic complexes, the rhodafurans, was achieved. Therefore, if the reaction of **29** with the silver salt is performed in the presence of a terminal alkyne, the insertion into the Rh=C bond takes place, forming the rhoda-1,3,5-hexatriene complexes **32–33** [35]. The *Z*-selectivity of complexes **32–33** would allow the intramolecular coordination of the oxygen to the rhodium nucleus, with the concomitant loss of CH_3_PF_6_ in order to obtain new rhodafuran complexes **34–35** (Scheme 11). These complexes represent one of the few examples of rhodafuran derivatives after Yamamoto and co-workers reported the reaction of RhCp*Cl_2_(PPh_3_) towards alkynes in the presence of KPF_6_ and water and Tani et al. described the reactivity of alkynes with rhodium carbonyl complexes [36,37].

These results support the need for the use of iridium as transition metal for the synthesis of these metallanaphthalene complexes. However, the adjustment of the methodology by including terminal alkynes allows for a new and versatile way of achieving the synthesis of rhodafurans with a broad range of substituents.

### 5.3. Effect of the Topology

In spite of the successful development of the methodology in the synthesis of iridanaphthalenes, the synthesis of larger structures is necessary in order to improve the properties of PAHs. However, the development of higher analogues of metallanaphthalene is a barely-studied field and only a few examples are known [32,33,34,38].

**Scheme 12:**
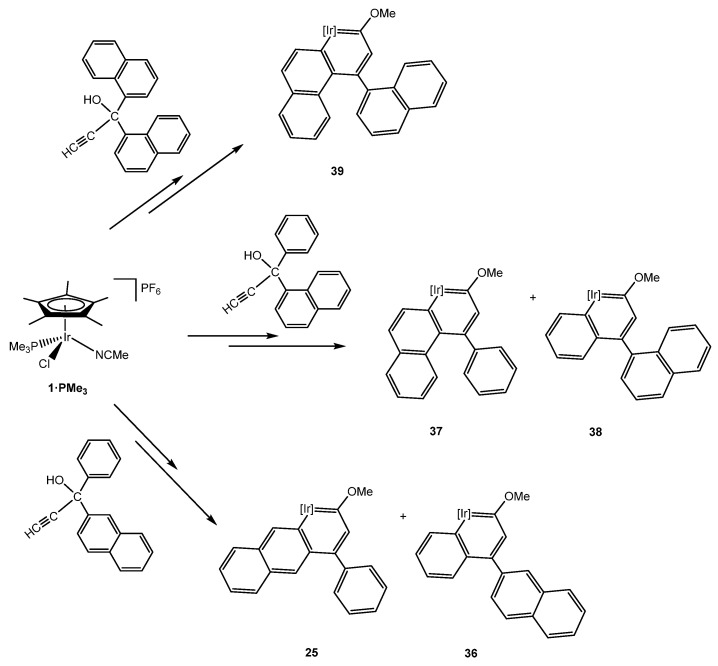
Synthesis of higher analogues [Ir] = [IrCp*(PMe_3_)]PF_6_.

In order to obtain higher analogues, such as iridaanthracene or iridaphenantrene derivatives, propargylic alcohols bearing at least one naphthyl group at *γ* position were synthesized. As was stated in Section 4, the *E*/*Z* isomerism at the methoxy(alkenyl)carbeneiridium complexes is crucial, however, the steric hindrance difference between a phenyl and a naphthyl substituents is not high enough to hamper the formation of both isomers and, consequently, the corresponding iridaaromatics (Scheme 12) [39].

**Figure 2:**
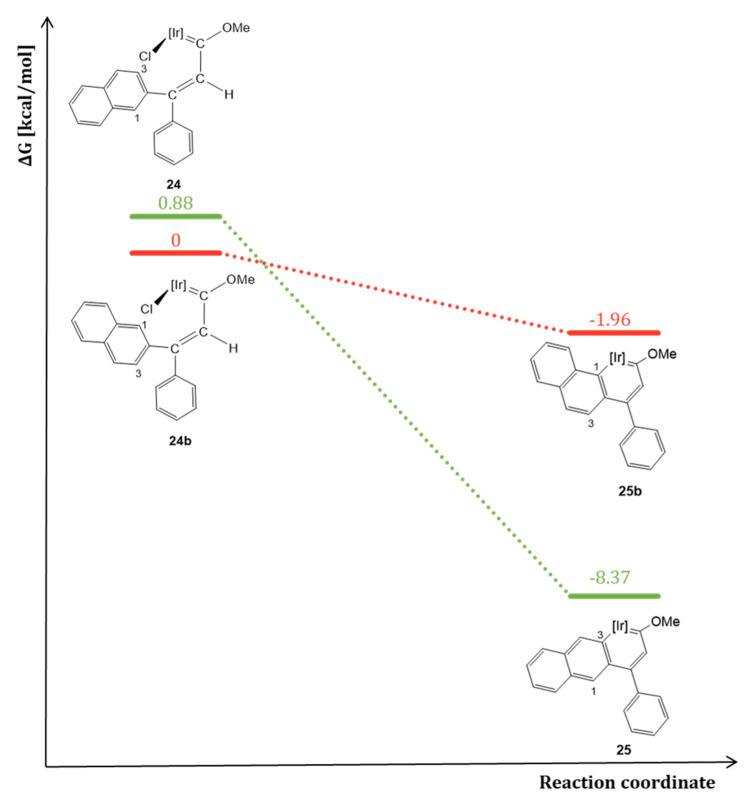
Energy profile for the formation of iridaanthracene complex **25** vs. iridaphenanthrene complex **25b** [Ir] = [IrCp*(PMe_3_)]PF_6_.

However, the naphthyl group can be bonded to the *γ* carbon through two different positions, leading to a variety of products. On the one hand, when coordinated at 2-position, an iridaanthracene complex **25** and the iridanaphthalene complex **36** were obtained (Scheme 12, bottom) [25]. Complex **25** represents the second example of a metallaanthracene described in literature. Frogley and Wright reported in 2016 the first metallaanthracene complex, also bearing iridium as metal center [32]. In contrast to the α-iridaanthracene complex **25**, Wright’s complex is a tethered-*β*-iridaanthracene. Although it was not possible to obtain X-ray data of **25**, its geometry optimization by DFT calculations showed that both iridaanthracene complexes show similar structural data which are also comparable to the purely organic anthracene. Regarding **25**, DFT calculations determined that two rotational isomers for the corresponding methoxy(alkenyl)carbene complex **24** were possible, due to the free rotation of the naphthyl substituent (Figure 2). This effect would lead to not only **25**, but also an iridaphenantrene derivative **25b**, which was never experimentally observed. Although DFT calculations showed that the most stable rotational isomer was the precursor of the iridaphenantrene complex, the energy difference of the isomers was too small (ΔG = 0.88 kcal/mol) compared to the higher stability of **25**, which is 6.41 kcal/mol more stable than **25b**, probably due to the larger steric hindrance of **25b** (Figure 2) [25].

On the other hand, the naphthyl group can be bonded at 1-position getting rid of the rotational isomerism.

Thus, despite the steric hindrance, activation at the C–H bond of the 1-naphthyl substituent led to the first iridaphenantrene complex **37** (Scheme 12, middle) [39]. Interestingly, the activation at the phenyl group gave rise to two diastereoisomers for the iridabinaphthyl complex **38**, observable by NMR spectroscopy, due to the hinder rotation about the biaryl bond as well as the iridium chiral center. This atropisomerism was also found in the iridaphenanthrene complex **39** formed by the activation of a symmetric propargylic alcohol bearing two 1-naphtyl substituents (Scheme 12, top) [39]. A theoretical study determined that the rotational barriers around the biaryl bond in **38** and **39**, as well as their aromaticity, are comparable to the 1,1′-binaphthalene and the calculated energy differences between *S*,*M* and *S*,*P* isomers can be related to the experimentally observed product ratios [39]. Prior to the formation of **37** and **39**, low temperature studies allowed for the characterization of the corresponding *η*^2^ intermediates, which in contrast to complex **23** (see Section 4), were capable of performing the C–H bond activation in order to obtain the iridaaromatic complexes.

**Scheme 13:**
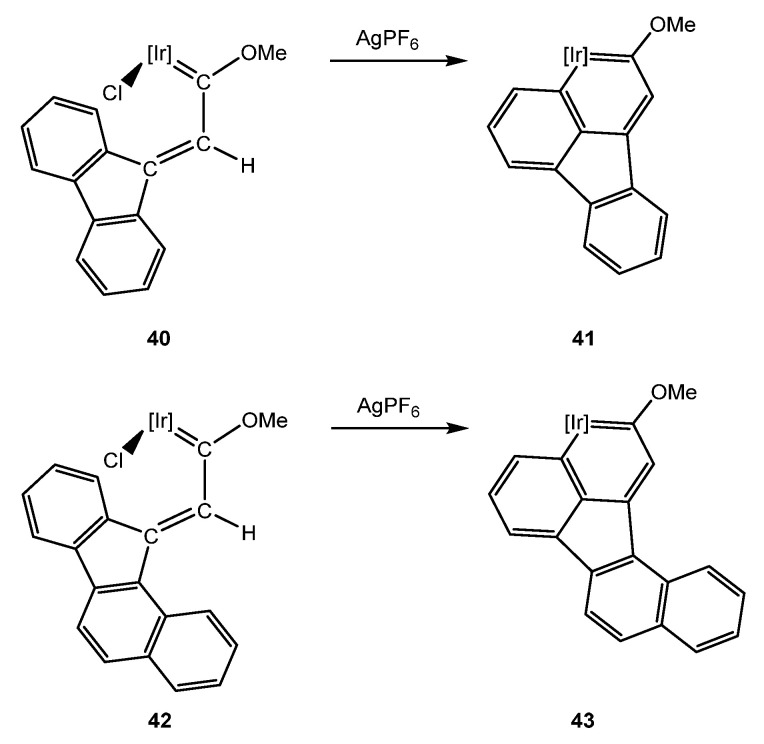
Synthesis of some iridafluoranthene derivatives. [Ir] = [IrCp*(PMe_3_)]PF_6_.

In addition, Liu et al. applied our methodology in order to obtain iridafluoranthene derivatives (Scheme 13) [33]. Thus, methoxy(alkenyl)carbene complexes, such as **40** and **42**, are capable of performing C–H bond activation in order to give the iridaaromatic complexes **41** and **43**, respectively. Interestingly, when an asymmetric propargylic alcohol is used, only one methoxy(alkenyl)carbene iridium complex **42** is obtained, and therefore, only one iridafluoranthene complex **43**. The authors proposed the unlikely activation of the remote fused ring to provide a seven-member metallacycle, which might be less favored than the obtained six-member ring [33]. This selectivity is consistent with our theoretical and experimental work using aromatic moieties at *γ* carbon with the *ortho* positions substituted (see Section 5.4) [40], as well as the effect of their steric hindrance, which would hinder the formation of the other *Z*-isomer.

The iridafluoranthene complexes showed a red-shift in the UV-Vis spectra for the higher conjugated derivatives such as **43**, while substitution at **41** did not have effect. In addition, CV measurements showed that only the first reduction was reversible. The substitution with electrondonating or electronwithdrawing groups at **41** caused a cathodic or anodic shift in the reduction potential, respectively, while **43** derivatives showed anode shift for the reduction and cathode shift for the oxidation potentials [33].

### 5.4. Special Systems

The chemistry of metallaaromatic complexes is not only limited to metallabenzenes, metallanaphthalenes or higher analogues [30,41,42,43,44,45]. As has been previously described by other groups, different topologies can produce properties which could be used in specific applications [30,43,46]. Therefore, the introduction of systems such as the spirobifluorene moiety (SBF) could be useful to generate new properties with optoelectronic applications.

**Scheme 14:**
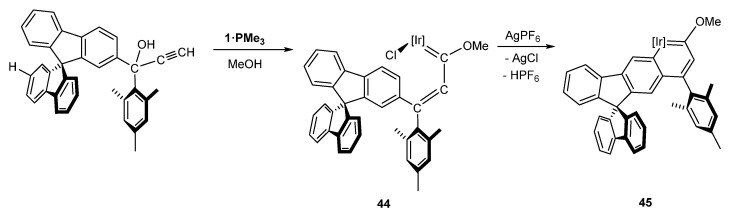
Spirobifluorene containing complexes. [Ir] = [IrCp*(PMe_3_)]PF_6_.

As described in Section 4, the higher steric hindrance of the SBF substituent with respect to the methyl group, was the key for the formation of the methoxy(alkenyl)carbene isomer **16** and, therefore, a five-membered metallacycle **17** upon C–H bond activation [24]. If the methyl group is substituted by a mesityl group, the *E* methoxy(alkenyl)carbene isomer with the SBF moiety near the iridium atom, **44**, is 1.85 kcal/mol more stable than the *Z* isomer [24], which leads to a selective synthesis of **44**. Subsequent activation of the C–H bond led to the first spirobifluorene metallaaromatic complex **45** (Scheme 14) [40].

Complex **45** showed interesting properties as a potential material in optoelectronics. It presented a high thermal stability above 473 K and higher aromatic character, as well as a hinder rotational barrier 20 times higher than biaryl systems **38** and **39**. In addition, a 1.1eV red-shift in the UV/Vis spectrum compared to the spirobifluorene was also observed [40].

## 6. Conclusions

In recent years our versatile synthetic methodology has allowed us to obtain different topologies of metallaaromatic complexes, such as iridanaphthalenes, iridaphenanthrenes, iridaanthracene, rhodafurans or the spirobifluorene iridanaphthalene.

The key aspect of our methodology is the precursor, a methoxy(alkenyl)carbene complex, which by C–H bond activation of one of the *ortho* carbons of an aromatic substituent on *γ* carbon leads to the formation of a metallaaromatic compound. In order to guarantee the success of this methodology, we have studied the influence of different substituents at the γ carbon, the metallic center, the substituents on the aromatic cycle and the possibility of using it to obtain different topologies. Therefore, it was concluded that: (1) it is necessary for one of the substituents on the *γ* carbon to possess a large steric hindrance in order to obtain a single *Z* or *E* isomer; (2) the existence of a second substituent, also aromatic, ensures the formation of the metallaaromatic complex due to its capability to delocalize the electronic density among the whole complex; (3) in this case, at least one of them need not be substituted in the *ortho* positions; (4) the increase of aromatic rings increases stability; (5) the most favored *ortho* position for C–H bond activation is the one that produces the least stressed cycle; and (6) the capacity of the methodology to synthesize different topologies is very broad.

All together gives the opportunity to synthesize analogues to polycyclic aromatic hydrocarbons, PMAHs, which are small fragments of graphene doped with a transition metal and thus to study their new properties and consequently their applications in the field of materials.
